# First Report of Swinepox in a Wild Boar in Italy: Pathologic and Molecular Findings

**DOI:** 10.3390/pathogens12030472

**Published:** 2023-03-16

**Authors:** Lisa Guardone, Katia Varello, Valeria Listorti, Simone Peletto, Lara Wolfsgruber, Roberto Zoccola, Vittoria Montemurro, Erika Messana, Elena Bozzetta, Pierluigi Acutis, Loretta Masoero, Elisabetta Razzuoli

**Affiliations:** Istituto Zooprofilattico Sperimentale del Piemonte, Liguria e Valle d’Aosta, Via Bologna 148, 10154 Torino, Italy

**Keywords:** infectious diseases, histopathology, vector-borne, pigs, wildlife, SWPV, molecular analysis

## Abstract

Swinepox virus (SWPV) is responsible for sporadic acute poxvirus infections in swine worldwide, causing a pathognomonic eruptive proliferative dermatitis. Beside direct and congenital transmission, the pig louse *Haematopinus suis* acts as a mechanical vector and favors virus infection through skin lesions. Infections are generally described in domestic pigs, while only a few cases have been reported in wild boars, in Austria and Germany. In September 2022, SWPV infection was suspected at *post-mortem* examination of a wild boar piglet with characteristic lesions in Liguria, Northwest Italy. The piglet was heavily parasitized by swine lice (*H. suis*). SWPV was then confirmed by histological and molecular analyses. Possible viral co-infections were also investigated (African swine fever virus, classical swine fever virus, parvovirus, circovirus, Aujeszky’s disease virus and hepatitis E virus). This article describes gross and histopathologic features of SWPV infection, differential diagnosis, and potential vector-borne transmission to domestic pigs, presenting a brief review of the literature on the topic. SWPV infection is reported in wild boars in Italy for the first time. The finding of SWPV in a wild boar in an area with a very limited pig population may suggest the existence of a “wildlife cycle” in the area. Further investigations are needed to understand the real risk of transmission of SWPV to domestic pigs as well as the role of other arthropod vectors.

## 1. Introduction

Swinepox virus (SWPV) is a globally distributed swine pathogen causing sporadic acute poxvirus infections in pigs, characterized by a pathognomonic eruptive proliferative dermatitis and secondary ulcerations [1]. Its main mode of transmission is through the biting of the pig louse *Haematopinus suis*, which acts as a mechanical vector; however, direct animal contact and congenital transmission have also been reported [2,3,4]. Swinepox was first described as a disease of domestic pigs in Europe in 1842 [5] and in the USA in 1929 [6], but outbreaks have subsequently been found in pigs also in Africa, Asia, Australia and South America [7,8,9,10,11,12,13]. It is commonly associated with poor sanitation [4], since the virus shows a high environmental stability and is very resistant to drying, as all poxviruses are [14].

Piglets up to 3 months of age are the most susceptible to the clinical disease, while adults usually develop a mild form of the infection [15]. The disease occurring after congenital infection seems to be more severe, with higher mortality rates [1,4]. Despite its wide distribution, studies on SWPV infection are scarce, maybe due to its self-limiting and sporadic nature [2,4]. However, knowledge about the prevalence, strain diversity, evolutionary origin and wildlife reservoirs of SWPV is limited [1].

SWPV consists of a 146 kbp linear double-stranded DNA genome and is the only member of the Suipoxvirus genus, which belongs the Chordopoxvirinae subfamily, within the Poxviridae family [2]. Due to its large genome, the SWPV has been used as a delivery vector for several bacterial and virus vaccines [2]. Clinical studies and experimental and in vitro infections have shown that SWPV displays a high degree of host specificity [1]. Among swine, infections are mainly described in domestic pigs, while only a few cases have been reported in wild boars, in Austria [16] and Germany [1]. 

In Italy, SWPV has been very sporadically reported in domestic pigs. Its presence has been demonstrated in Northern Italy since 2002 [17] and in central Italy since 2006 [18]. In November 2013, an outbreak affecting a group of 3-months old piglets was registered in an extensive organic farm holding around 110 animals of a local breed in Tuscany (Central Italy) [18]. The aim of this study is to report a SWPV infection in a wild boar piglet with characteristic lesions in Liguria region (Northwest Italy), describing gross and histopathologic findings, differential diagnosis, and potential vector-borne transmission to domestic pigs. The presented case represents the first description of SWPV in wild boars in Italy, suggesting that the virus is circulating in this host in the wild.

## 2. Materials and Methods

### 2.1. Post-Mortem Examination

In September 2022, a male striated wild boar piglet accidentally found dead (lat. 44.445309, long. 9.191845) was submitted by the local veterinary authority to the Istituto Zooprofilattico Sperimentale of Piedmont, Liguria and Aosta Valley (IZSPLV), section of Genoa, for necrospy and molecular testing for African Swine Fever (ASF), as part of the ongoing national passive surveillance activity. A complete *post-mortem* examination was conducted, and samples were taken for subsequent histopathologic analysis.

### 2.2. Histopathology

Cutaneous lesions (from neck and ear skin) and a submandibular lymph node were fixed in 10% neutral-buffered formalin, paraffine embedded, sectioned by a microtome at 4 µm and subsequently stained with haematoxylin and eosin (HE). The slides were evaluated under a light microscope (Zeiss Axio Scope.A1, Jena, Germany) at increasing magnification (10×, 20×, 40×). 

### 2.3. Molecular Analysis

Total DNA and RNA were extracted using a commercial virus extraction kit with Maxwell automatic extractor (Promega) from different anatomic samples: cutaneous lesions, submandibular and mesenteric lymph nodes, spleen, lungs, and liver. Molecular analyses for the detection of SWPV were conducted on cutaneous lesions, submandibular lymph node and spleen samples (Table 1). The SWPV amplicon was directly sequenced using PCR primers on a 3130XL Genetic Analyzer (Thermo Fisher Scientific Inc., Waltham, MA, USA). Sequences were aligned using the SeqMan software (Lasergene package. DNASTAR Inc., Madison, WI, USA) to obtain a consensus sequence and compared with available sequences retrieved from the National Center for Biotechnology Information (NCBI) database through the BLAST tool (http://blast.ncbi.nlm.nih.gov/Blast.cgi, accessed on 14 February 2023). Details on the other investigated viral infections and related tested samples are reported in Table 1. Negative and positive controls were used in every PCR reaction. Negative controls include an extraction control and an amplification control (water for molecular biology), positive controls consisted in reference material provided with the diagnostic kit (African Swine Fever) or by the National Reference Centre (Classical Swine Fever) or in DNA of field samples previously tested positive, with amplicons subjected to Sanger sequencing and confirmed by BLAST analysis.

## 3. Results

### 3.1. Post-Mortem Examination

**Figure 1:**
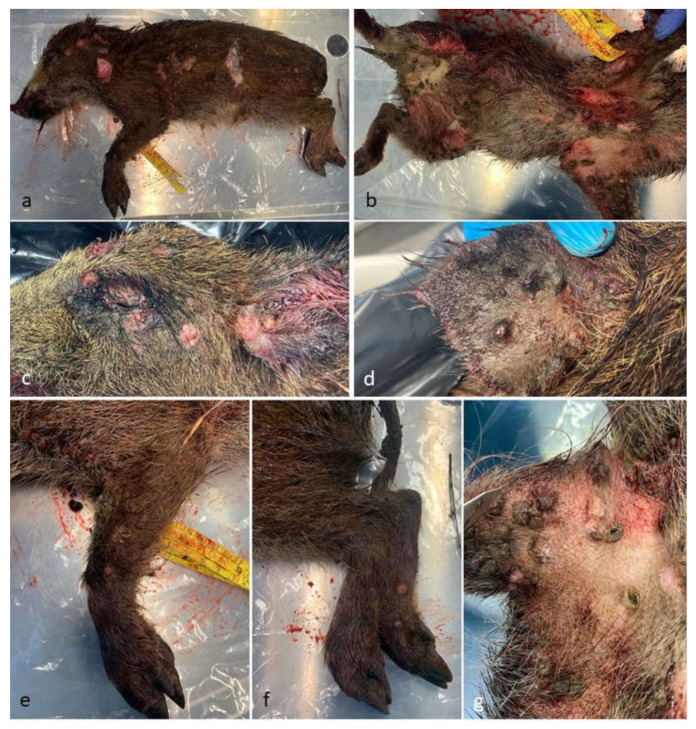
Gross aspect of the affected wild boar: (**a**) left flak; (**b**) ventral view; (**c**) detail of the head and periocular region; (**d**) detail of the pinna; (**e**,**f**) front and back legs; (**g**) axillary region.

The striated wild boar piglet (weight ~4 Kg) showed on external examination a heavy infection by the swine louse *H. suis*, a BCS of 2 and blood soiling on the snout. Macroscopically, multifocal to coalescing papular lesions (0.5–0.8 cm in diameter) were present on the skin of the snout, neck, torax, abdomen and paws, not involving the interdigital space (Figure 1). At necropsy, no apparent lesions affecting the abdominal viscera were observed, while, in the thorax, the lungs presented atelectasis affecting the apical pulmonary lobes. Traumatic lesions compatible with a car accident were found on examination of the head. The stomach was full of semi-digested material.

### 3.2. Histopathology

**Figure 2:**
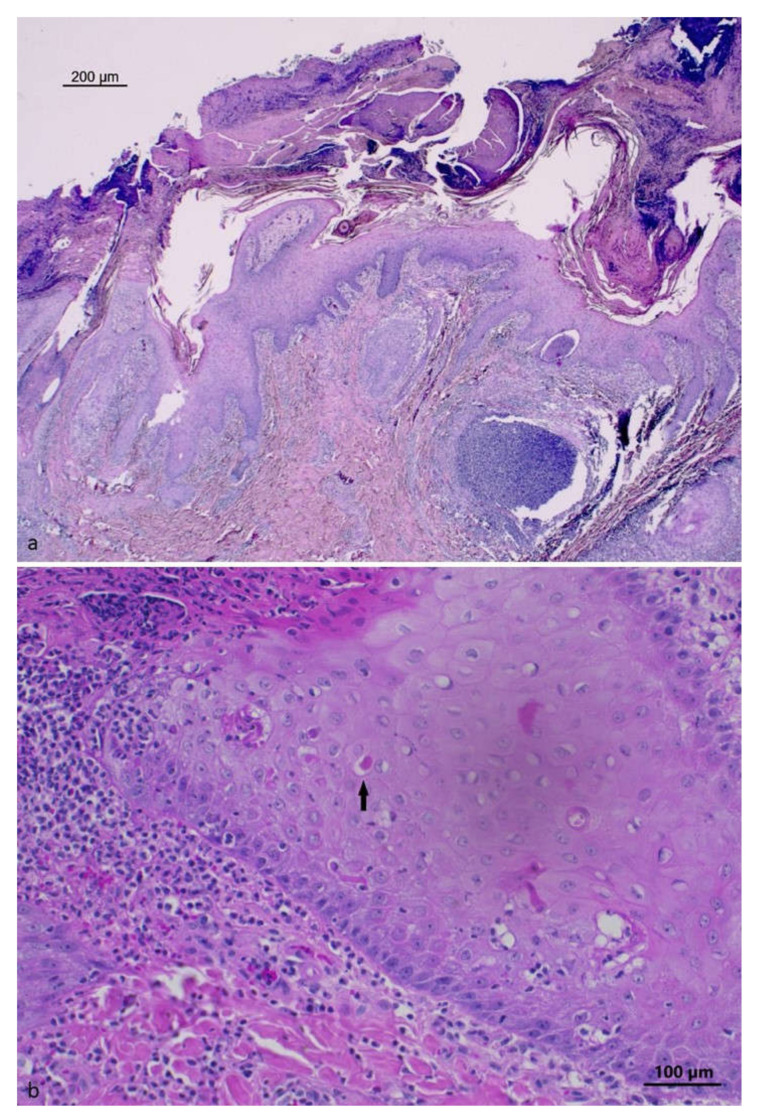
Skin, wild boar pinna: (**a**) severe pustular epidermitis with crusted areas (HE), Bar = 200 μm; (**b**) hyperplastic epidermis with eosinophilic inclusion body (black arrow) in the cytoplasm of keratinocytes (HE), Bar = 100 μm.

The histological examination of the skin lesions from the neck and ear showed areas of epidermal proliferation with foci of keratinocyte degeneration in the stratum spinosum, with rare intracytoplasmic eosinophilic inclusions. A severe multifocal infiltration of neutrophils was also observed, forming micro-abscesses at the intra-epidermal level with the formation of large surface crusted areas with the presence of bacterial aggregates. In some locations, the inflammatory process also involved adnexal structures. A severe and diffuse inflammatory infiltrate involving neutrophils, macrophages and lymphocytes in the dermis was also observed. The histopathologic picture is shown in Figure 2. 

The submandibular lymph node presented severe multifocal to coalescing inflammation characterized by intact and necrotic neutrophils with a large necrotic central area and the presence of bacterial aggregates.

### 3.3. Molecular Analysis

#### 3.3.1. SWPV

**Figure 3:**
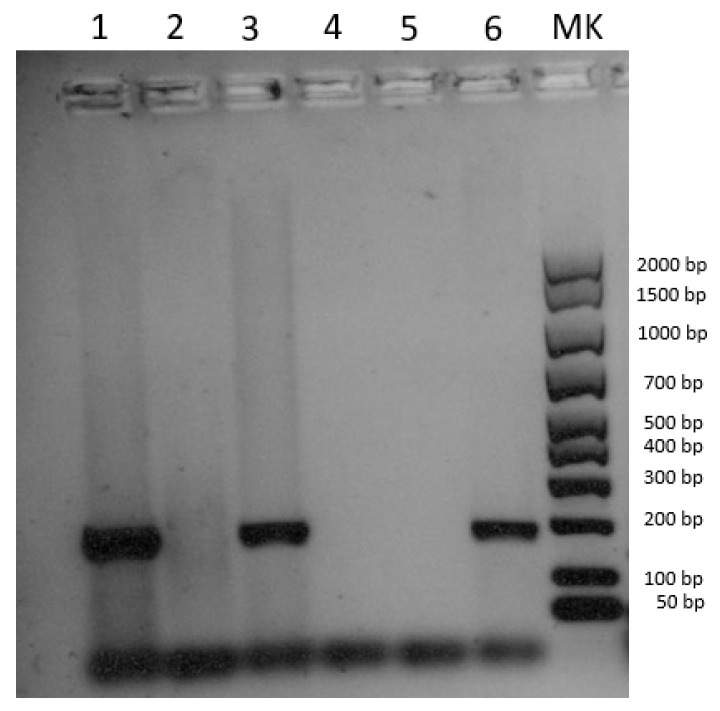
Electrophoresis gel image of the pan poxvirus PCR according to Li et al. [19]. Line 1: cutaneous lesions; line 2: spleen; line 3: mesenteric lymph nodes; line 4: negative extraction control; line 5: negative amplification control; line 6: positive control, DNA of Lumpy skin disease virus (Capripoxvirus).

DNA extracted from the cutaneous lesions and the submandibular lymph node tested positive for SWPV, while the spleen turned out negative (Figure 3). The positivity was confirmed by sequencing a 175 bp fragment of the putative metallo-protease gene. The SWPV sequence was submitted to NCBI GenBank under accession number OQ446858. Blast analysis revealed 100% identity with four sequences of the European–North American lineage (accession nrs. NC_003389, MZ682626, MZ773481, MZ773480) and 99.3% similarity with one sequence of the Indian lineage (accession nr. MW036632), according to the tentative classification recently proposed by Kumar et al. [25]. Since these five sequences are the only ones currently available for the target gene, with limited variability, a phylogenetic analysis was not carried out. 

#### 3.3.2. Other Viral Infections

Molecular analysis carried out to test for co-infections were all negative.

## 4. Discussion

### 4.1. Gross and Histopathologic Features of Swinepox Virus Infection

The macroscopic aspect of SWPV infection has been described as a multifocal, eruptive dermatitis, commonly affecting the abdomen, inner surface of the legs, pinnae and occasionally the snout, vulva and back [9,10,13]. The development of a generalized disease, with lesions affecting the whole body, has been reported for suckling piglets [2], as observed in the described case. It has been hypothesized that the main lesions, observed on the abdomen and inner surface of the legs, including the udder and vulva, correspond to the predilection sites of the pig louse [1]. Secondary bacterial infections, facilitated by SWPV infection and disruption of skin epithelium, lead to more severe lesions and formation of local abscesses [2,10]. According to the literature, clinical lesions are generally restricted to the skin, with occasional mild changes in the superficial lymph nodes [1]. In a study describing congenital infection in 14 piglets, pustular and ulcerative lesions were also found on the tongue and hard palate of four piglets, while no significant lesions were found on the internal organs [4]. No internal lesions were also found in an outbreak in Papua New Guinea [8]. These observations agree with our results, as we only observed typical lesions at the skin level, not involving internal organs. 

The skin lesions typically start with petechiae, which may appear 2 days post-infection, and then evolve to papules, and to pustules that eventually originate crusts (scabs) after a week. A true vesicle stage is absent or transient [26]. The crusts ultimately shed, leaving skin discoloration [2]. The infection is usually self-limiting, with resolution in 3–4 weeks [27], although secondary bacterial infection prolongs the duration of symptoms [4]. A transient rise in temperature and appetite loss may occur. The infected pigs may exhibit conjunctivitis, unilateral or bilateral keratitis and/or pan-ophthalmia. Kerato-conjunctivitis without cutaneous eruption lesion has also been observed [15]. It is not fully understood how spreading from the primary site of replication to other parts of the organism occurs, as evidenced by the fact that viremia is lacking and SWPV could not be isolated from the blood of infected animals [28,29]. This could explain the negative PCR result obtained for DNA extracted from the spleen in our study.

The most readily visible histological changes of tissues from swinepox-infected pigs include hydropic degeneration of the epidermal stratum spinosum and keratinocytes, where viral replication occurs [1]. Eosinophilic, rounded, intracytoplasmic inclusion bodies 3–8μm in diameter can be observed in keratinocytes [9,12]. Indeed, the histological analysis of the lesions from the neck and ear skin of the described case showed keratinocyte degeneration at this level, with intracytoplasmic eosinophilic inclusions (Figure 2). Traditionally, the diagnosis of swinepox is based on the observation of the typical clinical signs and lesions, and on the observation of microscopic characteristics [30]: infected cell cytoplasm is enlarged and contains inclusion bodies, whereas the nucleus exhibits margination of chromatin and a large, central “vacuole” [2]. The secondary bacterial infection can complicate the histologic picture [8]. Histopathologic changes caused by SWPV are very similar to those of other poxviruses, which however are host specific.

The differential diagnosis of swinepox includes vaccinia virus (VACV) infection, vesicular diseases (including foot and mouth disease), parvovirus, pityriasis rosea, parakeratosis, parasitic skin disorders (including sarcoptic mange and Acarus (*Tyroglyphus*) spp. mite irritation), allergic skin reactions, insects’ bites, early stages of ringworm, thrombocytopenic purpura, localized staphylococcal or streptococcal epidermitis and cutaneous erysipelas [2,4,9,29]. The multispecies-infecting VACV is the etiological agent of the most similar disease, although lesions are smaller and the incubation period shorter. VACV still circulates in some countries, such as Brazil, but not in Western Europe and can be easily differentiated from SWPV by molecular tools [1].

### 4.2. Potential Vector-Borne Transmission

SWPV enters the host through a break in the skin, replicates in the cytoplasm of keratinocytes of the stratum spinosum [29], and it is shed from nasal and oral secretions and skin lesions. It is present in infected epithelium and in dry scabs produced in the later stages of the infection. Poxviruses in general show a high environmental stability and remain contagious over a period of several months. They are particularly resistant to drying, a feature which is further enhanced by the organic materials in which they are released into the environment, such as crusts [14]. Abraded skin can serve as the route of entry for the virus [15]. As mentioned, in addition to direct contact between infected and susceptible animals and to congenital transmission, insect vectors such as *H. suis* have been shown to facilitate viral spread between populations [31]. Early experiments investigated the role of *H. suis* in swinepox infections and demonstrated its function as a mechanical vector, but not as an intermediate host [3]. Flies and mosquitoes also seem to be implicated as mechanical vectors [2,15,29]. However, studies on vector-borne transmission are scanty and outdated.

In the most recently described outbreak in Italy, the source of SWPV infection could not be demonstrated, but a role in the transmission of the disease within the farm was attributed to *H. suis,* which was parasitizing the animals [18]. In two outbreaks in the northeast of India, the entire body of the affected animals was found to be heavily infected with swine lice [7]. Similarly, in five swinepox outbreaks in northeastern Brazil, affected backyard pigs from herds with poor hygienic conditions presented with severe fly and lice infestations [12]. In contrast, in an outbreak described in Papua New Guinea, lice were not observed, but a large number of stable flies, *Stomoxys calcitrans*, was present. Their role as a mechanical vector had already been hypothesized for fowl pox. Organophosphate insecticides were sprayed at the beginning of the outbreak to control the insects [8]. Moreover, in an outbreak described in Australia, sucking lice were not found, but mosquitoes, biting flies and midges were abundant in the affected environment. In such a case, pox-carrying insects could have been carried by the winds [9] and the spreading of SWPV could occur over longer distances. 

In this study, SWPV infection in wild boars in Italy was reported for the first time, and only a few other reports in this wild host are known from the literature [1,16]. In a recent study conducted in Germany, SWPV strains isolated from a wild boar piglet and a congenitally infected domestic piglet were sequenced, finding only 0.076% divergence [1]. Interestingly, domestic pig breeding is traditionally very limited in Liguria, and the swine population has been further reduced due to the restrictions following the ASF outbreak, which started in January 2022. In contrast, the wild boar population is abundant and rising (authors’ note). Thus, it can be hypothesized that SWPV may be present in wild boars in the area, circulating in a wildlife (sylvatic) cycle. Although strict sanitary measures and high hygienic standards are usually implemented in industrialized countries to protect the livestock population against contact with pathogens present in the environment and to prevent potential direct or indirect contacts with wild boar, vector-borne transmission may be more challenging to control, especially in extensive farming. Further investigations are needed to understand if SWPV is present in wild boars in other geographical areas, as well as the real risk of transmission to domestic pigs, and the role of *H. suis* and other arthropod vectors.

## Figures and Tables

**Table 1:** Molecular analyses conducted to search for swinepox virus (SWPV) and co-infections and their results. NA: Not Available, as covered by industrial copyright.

Etiological Agent	Method	Tested Sample
Swinepox virus	• End-point PCR (2720 Thermal cycler, Life Technologies) • Primers: ACACCAAAAACTCATATAACTTCT; CCTATTTTACTCCTTAGTAAATGAT (Li et al. [19]) • Target gene: 220 bp covering the insulin metalloproteinase-like protein gene and the intracellular mature virion membrane protein gene • PCR mixture: 1X Platinum qPCR SuperMix-UDG (Invitrogen), 0.1 µM of each primer and 2 µL of the template, in a total volume of 20 µL • PCR cycling conditions: 95 °C/5 min, followed by 40 cycles at 95 °C/30 s and 68 °C/60 s • Amplification products loaded onto agarose gel (1.8%) and compared to the Amplisize Molecular Ruler (Biorad)	Skin lesions Submandibular lymph node Spleen
African Swine Fever virus	• Real-time PCR (CFX96 Biorad) • Primers and probe: NA • Target gene: virion capsid protein p72 encoded by the B646L gene • PCR mixture: ID Gene™ African Swine Fever Duplex kit (IDVet) following the manufacturer’s protocol • PCR cycling conditions: 95 °C/10 min, followed by 40 cycles at 95 °C/15 s and 60 °C/60 s	Spleen
Classical Swine Fever virus	• Real-time PCR (CFX96 Biorad) • Primers and probe: CSF100-F: ATGCCCAYAGTAGGACTAGCA; CSF192-R: CTACTGACGACTGTCCTGTAC; CSF-P: TGGCGAGCTCCCTGGGTGGTCTAAGT (Hoffman et al. [20]) • Target gene: 93 bp pf the 5′ non-translated region • PCR mixture: 4X Reliance One Step multiplex Supermix (Biorad), 0.2 µM of each primer and 5 µL of the template, in a total volume of 20 µL • PCR cycling conditions: 50 °C/10 min, 95 °C/10 min, followed by 40 cycles at 95 °C/10 s and 60 °C/30 s	Spleen
Porcine Parvovirus (PPV)	• End-point PCR (2720 Thermal cycler, Applied Biosystems) • Primers: PPV-F: CACAGAAGCAACAGCAATTAGG; PPV-R: CTAGCTCTTGTGAAGATGTGG (Ogawa et al. [21]) • Target gene: 203 bp of the VP2 gene • PCR mixture: 1X Buffer of Taq Platinum DNA polymerase (Invitrogen), 0.05 U/µL of Taq polymerase, 3 mM of MgCl2, 200 µM of dNTP, 1µM of each primer and 2 µL of the template, in a total volume of 25 µL • PCR cycling conditions: 94 °C/1 min, followed by 35 cycles at 94 °C/30 min, 53 °C/90 s and 72 °C/90 s; finally, 72 °C/10 min • Amplification products were loaded onto agarose gel (1.8%) and compared to the Amplisize Molecular Ruler (Biorad)	Mesenteric lymph node
Porcine circovirus type II (PCV type II)	• End-point PCR (2720 Thermal cycler, Applied Biosystems) • Primers: CF8: TAGGTTAGGGCTGTGGCCTT; CR8: CCGCACCTTCGGATATACTG (Larochelle et al. [22]) • Target gene: 263 bp fragment of the ORF2 region • PCR mixture: 1x Buffer of AmpliTaq Gold DNA polymerase (Thermofisher Scientific), 0.05 U/µL of Taq polymerase, 1.5 mM of MgCl_2_, 200 µM of dNTP, 1µM of each primer and 2 µL of the template, in a total volume of 50 µL • PCR cycling conditions: 95 °C/15 min, 35 cycles of denaturation at 95 °C/20 s, annealing at 55 °C/30 s and extension at 72 °C/30 s and a final extension of 72 °C/5 min	Mesenteric lymph node
Aujeszky’s disease virus	• Real-time PCR (CFX96 Biorad) • Primers and probe: gB718F: ACAAGTTCAAGGCCCACATCTAC; gB812R: GTCYGTGAAGCGGTTCGTGAT; gB785P: ACGTCATCGTCACGACC (Ma et al. [23]) • Target gene: glycoproteins B gene • PCR mixture: 1X Platinum qPCR SuperMix-UDG (Invitrogen), 0.4 µM of each primer, 0.2 µM of the probe and 2.5 µL of the template, in a total volume of 25 µL • PCR cycling conditions: 50 °C for 2 min, 95 °C for 15 min, followed by 45 cycles at 94 °C for 15 s and 62 °C for 60 s	Lungs
Hepatitis E virus (HEV)	• Real-time PCR (CFX96 Biorad) • Primers and probes: JVHEVF: GGTGGTTTCTGGGGTGAC; JVHEVR: AGGGGTTGGTTGGATGAA; JVHEVP: TGATTCTCAGCCCTTCGC (Jothikumar et al. [24]). • Target gene: ORF3 region • PCR mixture: 1X Reaction mix of SuperScriptIII Platinum One-Step qRT-PCR System (Invitrogen), 0.5 µL of Taq mix, 1 mM of MgSO4, 0.25 µM of each primer, 0.1µM of the probe and 3 µL of the template, in a total volume of 25 µL • PCR cycling conditions: 50 °C for 30 min and 95 °C for 15 min followed by 45 cycles at 95 °C for 10 s, 55 °C for 20 s and 72 °C for 15 s	Liver

## Data Availability

All data deriving from the study are given in the article text.
